# Novel Online Platform for Trauma Care—Integrating Trauma Phenotypes to Optimize the Trauma and Injury Severity Score Model: Retrospective Cohort Study

**DOI:** 10.2196/90011

**Published:** 2026-06-02

**Authors:** Jotaro Tachino, Shigeto Seno, Hisatake Matsumoto, Tetsuhisa Kitamura, Shunichiro Nakao, Hiroshi Ogura, Jun Oda

**Affiliations:** 1 Department of Traumatology and Acute Critical Medicine Graduate School of Medicine The University of Osaka Suita City, Osaka Japan; 2 Department of Bioinformatic Engineering Graduate School of Information Science and Technology The University of Osaka Suita City, Osaka Japan; 3 Division of Environmental Medicine and Population Sciences, Department of Social and Environmental Medicine Graduate School of Medicine The University of Osaka Suita City, Osaka Japan

**Keywords:** prognostication, critical care, artificial intelligence, phenotyping, clinical decision support, Trauma-Vis

## Abstract

**Background:**

Severe trauma remains a leading cause of admission to the intensive care unit. The Trauma and Injury Severity Score (TRISS) is an established standard for predicting outcomes and benchmarking the quality of trauma care globally. However, the TRISS model has some limitations when used for benchmarking trauma care.

**Objective:**

This study aimed to determine whether machine learning–derived trauma phenotypes can complement the TRISS via multivariable modeling to improve in-hospital death prediction. We also introduce “Trauma-Vis,” a freely accessible web-based platform, to facilitate the availability of this integrated assessment approach to clinicians.

**Methods:**

In this retrospective cohort study using the nationwide Japan Trauma Data Bank (JTDB), which encompasses data from 303 hospitals in Japan, we divided the data chronologically into a derivation cohort (JTDB 2015-2018) and a temporal validation cohort (JTDB 2019-2022). An integrated model was developed using multivariable logistic regression, incorporating the logit-transformed TRISS-predicted mortality and the assigned trauma phenotypes. After applying the exclusion criteria, 87,882 patients with blunt trauma were analyzed in the derivation cohort and 80,964 in the validation cohort. Predictive performance was evaluated using the area under the receiver operating characteristic curve, Brier score, logarithmic loss, net reclassification improvement, integrated discrimination improvement, and decision curve analysis.

**Results:**

In the derivation cohort, multivariable modeling demonstrated that trauma phenotype classification significantly recalibrated mortality risk; multiple phenotypes exhibited significant independent associations with in-hospital death after adjusting for baseline TRISS predictions (eg, for phenotype 8: odds ratio 2.38, 95% CI 2.11-2.68; *P*<.001). In the temporal validation cohort, the integrated multivariable model yielded higher performance metrics than the baseline TRISS model: the area under the receiver operating characteristic curve increased from 0.889 to 0.897 (DeLong test, *P*<.001), Brier score improved from 0.0454 to 0.0394, and logarithmic loss decreased from 0.1670 to 0.1458. The integrated model demonstrated a calibration intercept of –0.152 and a slope of 0.965 and provided a higher net benefit in the decision curve analysis across evaluated threshold probabilities.

**Conclusions:**

Integrating machine learning–derived trauma phenotypes with the TRISS via multivariable modeling improved the accuracy and utility of in-hospital death prediction. The developed “Trauma-Vis” platform demonstrates the technical feasibility of providing real-time risk stratification at the bedside.

## Introduction

Severe trauma remains a leading cause of admission to the intensive care unit (ICU), and accurate survival prediction remains a critical challenge at the bedside. The Trauma and Injury Severity Score (TRISS) is an established standard for predicting outcomes and benchmarking the quality of trauma care globally [1,2]. While primarily designed for post hoc evaluation of trauma system performance, these scores implicitly influence clinical prognostication, resource allocation decisions in the ICU, and interpretation of clinical trial results.

Since its development in the 1980s, the TRISS has shown several limitations, including poor evaluation of physiological reserve, challenges in handling missing values, insufficient calibration, and outdated weighting [3,4]. Moreover, the parameters included in the TRISS model are limited and some variables are categorized, leading to information loss. Additionally, parameter nonlinearity [5] and interactions are not considered [4], and specific combinations of injured regions have been suggested to interact, affecting trauma mortality [6,7]. Furthermore, the coefficients in the TRISS model have not kept pace with advances in medical technology and treatment methods. Consequently, the model’s prediction accuracy has decreased in mature trauma systems [8,9], complicating trauma center performance benchmarks [9].

Evaluating the performance of clinical prediction models is essential for maintaining predictive accuracy in target patient populations and specific environments [10]. Despite the presence of standardized TRISS revisions [11,12] and machine learning approaches [13-19], developing models that are both accurate and clinically practical at the bedside remains challenging [20].

A key concept in modern critical care is precision medicine, including enrichment strategies to optimize clinical trials and treatment [21]. These strategies aim to improve therapeutic development by targeting specific patient populations, especially in heterogeneous cohorts seen in ICUs. Prognostic enrichment can identify populations requiring therapeutic intervention by identifying subgroups likely to have poor outcomes [22]. Previous studies that applied machine learning methods represent a novel approach to identifying potential subphenotypes of trauma [23-25]. In trauma phenotyping, the variables included in the TRISS model are used to their full potential (eg, as continuous values) and combined with other factors, such as comorbidities, allowing mortality risk to be captured from a different perspective than TRISS alone. The aforementioned studies identified high-mortality phenotypes, suggesting their potential use for prognostic enrichment in trauma care. However, this phenotyping approach has thus far been limited to research reports and has not yet been translated into a real-time, clinically deployable patient assessment platform.

We hypothesized that integrating machine learning–derived trauma phenotypes with the TRISS model would enable more precise outcome predictions. Therefore, we aimed to assess the ability of these phenotypes to complement the TRISS model in identifying high-risk subgroups, using a large-scale national trauma registry, and to introduce “Trauma-Vis,” a freely accessible online platform that makes this integrated phenotype assessment available to front-line clinicians.

## Methods

### Study Design and Outcome

This retrospective cohort study was a secondary analysis of data from the Japan Trauma Data Bank (JTDB), a nationwide trauma registry that prospectively collects comprehensive trauma data from 303 participating tertiary emergency medical facilities across Japan (as of March 2022; Multimedia Appendix 1). Data were collected from January 1, 2015, to December 31, 2022.

We evaluated the baseline performance of the TRISS model and assessed its complementary value when integrated with previously developed trauma phenotypes. The primary outcome was in-hospital death. The study design and data analysis process adhered to the Transparent Reporting of a multivariable prediction model for Individual Prognosis or Diagnosis (TRIPOD) guidelines [26] and the Strengthening the Reporting of Observational Studies in Epidemiology (STROBE) guidelines [27].

### Ethical Considerations

This retrospective cohort study was conducted in accordance with the Declaration of Helsinki and approved by the Ethics Committee of the University of Osaka (institutional review board approval number 16260-4). The requirement for informed consent regarding registration in the JTDB, retrospective analysis of anonymized data, and publication of results was waived following approval from the Japanese Association for the Surgery of Trauma and ethics committees of the participating institutions in accordance with the ethical guidelines for medical and health research involving human participants issued by the Ministry of Health, Labor, and Welfare of Japan. To protect patient privacy and confidentiality, all study data from the JTDB were provided by the Japan Trauma Care and Research in an already anonymized and deidentified format. No financial compensation was provided to the human participants whose data were included. The developed “Trauma-Vis” platform is a web-based application where input clinical variables are processed temporarily on the server for real-time calculation. No patient data are persistently stored, logged, or shared with third parties. No identifiable images of individual participants are included in this paper.

### Participant Selection

The study population comprised all patients with blunt trauma registered in the JTDB. Registration criteria included patients transported to participating facilities with suspected injuries and an Abbreviated Injury Scale (AIS) score ≥3 [28].

The exclusion criteria were indirect transport, Injury Severity Score (ISS) of 75 [29], cardiac arrest upon hospital arrival, unknown age or sex, and unknown outcomes. These selection criteria were consistent with those used in a previous study that identified trauma phenotypes [23].

### Variables and Missing Data

Patient data were collected from the JTDB, including age, sex, comorbidities, injury mechanism, vital signs at initial assessment, AIS codes, transfusion data, treatment details, and clinical outcomes. Follow-up was continued until discharge or death. Consistent with standard trauma registry practices, blank AIS codes were deterministically treated as “0” (no injury). Prior to the clustering analysis, we assessed the proportion of missing data across all 14 variables selected for the model. To handle missingness, we applied a random forest–based imputation procedure using the “missForest” package [30]. Importantly, the imputation was performed independently for each cohort; the missForest algorithm was fitted and applied separately to the derivation cohort (2015-2018) and the validation cohort (2019-2022), ensuring that no information from one cohort influenced the imputation in the other. This imputation was strictly limited to physiological variables (eg, vital signs and Glasgow Coma Scale score). Anatomical injury variables, including AIS codes, were not subjected to any imputation.

### Trauma Phenotype Mapping and Platform Implementation

Before phenotyping, correlation coefficients between the variables were examined to avoid multicollinearity. Trauma phenotypes were assigned using a fixed *k*-nearest neighbors model (k=5) previously trained on JTDB data (January 2013-June 2015; n=42,780) [23]. The model classified patients into 8 phenotypes based on 14 standardized variables (demographics, vital signs, comorbidities, and AIS scores). While our previous biological profiling subdivided the high-mortality phenotype into 4 subphenotypes (clusters 8-11) [23], this study retains the original 8-phenotype classification. Consistent with our previous clinical studies, including those on tranexamic acid [24,25], we avoided further subdivision to preserve statistical power. Furthermore, rapid identification of the overarching high-mortality phenotype is clinically sufficient to guide acute management, making the 8-cluster model both statistically robust and practically actionable.

For platform implementation in “Trauma-Vis” [31], a workflow diagram of the phenotype assignment process and multivariable probability calculation pipeline is provided in Figure S1 in Multimedia Appendix 1. The system calculates cluster probabilities using 2-dimensional kernel density estimation and displays patient positioning using uniform manifold approximation and projection [32]. Additionally, to identify similar historical cases, the platform calculates Euclidean distances between the standardized input data and all samples in the derivation cohort, selecting 50 nearest neighbors. The platform concurrently calculates established trauma scores (ISS, Revised Trauma Score [RTS] [1], TRISS, and reverse shock index multiplied by the Glasgow Coma Scale score [rSIG] [33]).

### Statistical Analysis

Patient characteristics were described using medians (IQRs) and frequencies (percentages). For model development and validation, the data were chronologically divided into a derivation cohort (JTDB 2015-2018) and a validation cohort (JTDB 2019-2022). Baseline TRISS-predicted survival probabilities were calculated using the blunt trauma coefficients published by Boyd et al [2]: intercept b0=−0.4499, b1=0.8085 for the RTS, b2=−0.0835 for the ISS, and b3=−1.743 for the age index. The standard TRISS probability of survival (Ps) was first converted to a predicted mortality probability (P_mortality=1−Ps), which was then logit-transformed (logit(P_mortality)=ln[P_mortality/(1−P_mortality)]) to provide a continuous, additive covariate for the multivariable logistic regression. The binary age index was defined as 0 for patients younger than 55 years and 1 for patients aged 55 years or older. To appropriately recalibrate mortality risk without artificially distorting continuous probability outputs, we developed a mathematically principled integrated model using multivariable logistic regression. Specifically, we constructed a model predicting in-hospital death using the logit-transformed baseline TRISS-predicted mortality probability and the assigned trauma phenotypes (as categorical variables) as independent covariates. The derivation cohort was used to evaluate the baseline performance of the TRISS model and to train the integrated multivariable logistic regression model. Subsequently, the developed model was applied to the validation cohort to appropriately demonstrate its forward-facing temporal predictive validity. To evaluate the added prognostic value of integrating trauma phenotypes without introducing circular logic, we assessed model predictive performance based solely on the raw binary outcome of in-hospital death. Specifically, we constructed a model predicting raw binary in-hospital mortality using the logit-transformed TRISS-predicted mortality probability and assigned trauma phenotypes (encompassing all 8 phenotypes, as a categorical variable) as independent covariates. Multicollinearity among covariates was assessed using the variance inflation factor. This multivariable approach ensures that the continuous predicted probability for every patient is automatically recalibrated according to the specific odds ratio of their assigned phenotype, rather than applying a manual threshold-based adjustment to a single subgroup. We compared the baseline TRISS model against the integrated model using the area under the receiver operating characteristic (AUROC) curve, accompanied by the DeLong test. To evaluate classification accuracy, sensitivity, and specificity were calculated using a predicted mortality probability threshold of 0.5. Overall model performance and calibration were quantitatively evaluated using the Brier score, logarithmic loss (LogLoss), calibration intercept, and slope. Furthermore, to quantify the incremental value in predictive accuracy, we calculated the continuous net reclassification improvement and integrated discrimination improvement (IDI) [34]. To evaluate the clinical utility of the models for real-time decision support, we performed a decision curve analysis (DCA), which calculates the net benefit across a range of threshold probabilities [35]. Analyses were performed using R software (version 4.5.2; R Core Team).

## Results

### Study Population

**Figure 1:**
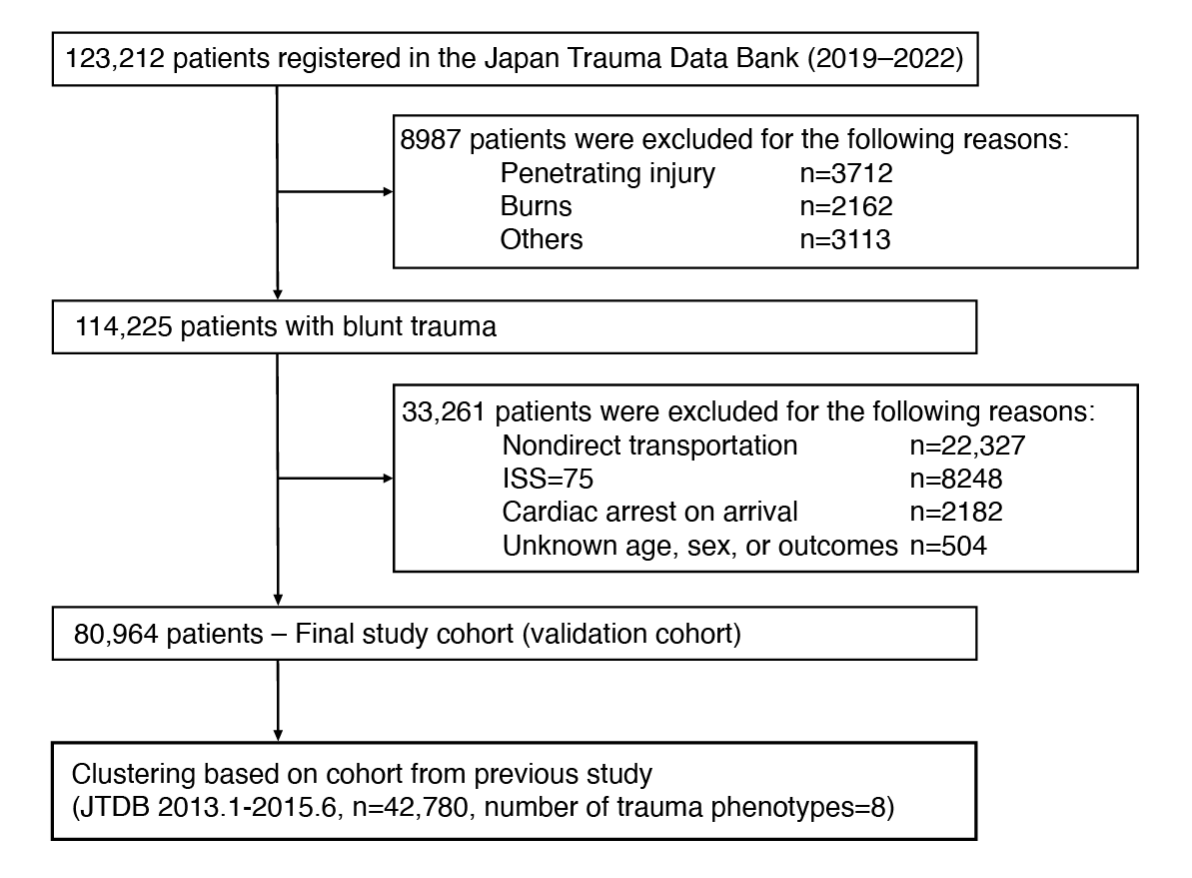
Patient selection flowchart for the temporal validation cohort. This flowchart details the inclusion and exclusion criteria applied in this retrospective cohort study. The validation cohort consists of patients with blunt trauma registered in the Japan Trauma Data Bank (JTDB) across 303 tertiary emergency medical facilities in Japan between 2019 and 2022. The final step illustrates the application of the pretrained k-nearest neighbors clustering algorithm, which was derived from a historical JTDB dataset (January 2013 to June 2015), to assign the 8 trauma phenotypes. ISS: Injury Severity Score; JTDB: Japan Trauma Data Bank.

For the derivation cohort (2015-2018), 141,743 patients were registered. After applying the exclusion criteria, 87,882 patients with blunt trauma were analyzed (Figure S2 in Multimedia Appendix 1). Correlation coefficients between the variables confirmed the absence of severe multicollinearity (Figure S3 in Multimedia Appendix 1). Baseline characteristics, variable distributions across phenotypes, and TRISS calibration performance in the derivation cohort are provided in Tables S1 and S2 and Figures S4 and S5 in Multimedia Appendix 1. For the validation cohort (2019-2022), 123,212 patients were registered. After excluding 8987 patients with nonblunt trauma, 114,225 patients with blunt trauma remained. Of these, 33,261 patients met the predefined exclusion criteria, leaving 80,964 patients for the final analysis (Figure 1).

The median age was 70 (IQR 48-82) years, the median ISS was 10 (IQR 9-18), and the in-hospital mortality rate was 6% (4883/80,964). Baseline characteristics and phenotype distributions for the validation cohort are presented in Table 1 and Figure S6 in Multimedia Appendix 1.

**Table 1:** Baseline characteristics of patients in the validation cohort (Japan Trauma Data Bank 2019-2022).

Characteristics^a^		Trauma phenotype 1 (n=9556)	Trauma phenotype 2 (n=1253)	Trauma phenotype 3 (n=4308)	Trauma phenotype 4 (n=16,060)	Trauma phenotype 5 (n=20,426)	Trauma phenotype 6 (n=5236)	Trauma phenotype 7 (n=19,358)	Trauma phenotype 8 (n=4767)	Overall (N=80,964)
Age (years), median (IQR)		54 (33-72)	62 (40-77)	81 (73-87)	48 (26-67)	82 (73-89)	54 (31-73)	71 (54-81)	70 (50-81)	70 (48-82)
Sex (male), n (%)		6484 (67.9)	907 (72.4)	2125 (49.3)	12,894 (80.3)	5310 (26)	3804 (72.7)	13,541 (70)	3192 (67)	48,257 (59.6)
Number of comorbidities, median (IQR)		0 (0-0)	0 (0-1)	3 (3-5)	0 (0-0)	0 (0-1)	0 (0-0)	0 (0-1)	0 (0-1)	0 (0-1)
Respiratory rate (/min), median (IQR)		22 (18-26)	20 (17-24)	19 (16-22)	21 (18-25	19 (16-21)	20 (18-24)	19 (16-22)	20 (16-24)	20 (17-24)
Heart rate (bpm), median (IQR)		88 (75-102)	87 (74-101)	83 (72-95)	87 (76-100)	80 (71-91)	87 (74-101)	80 (70-91)	93 (75-112)	83 (72-96)
Systolic blood pressure (mm Hg), median (IQR)		126 (106-145)	130 (109-153)	149 (129-169)	134 (117- 151)	151 (132-170)	137 (119-158)	145 (126-167)	138 (105-169)	141 (121-162)
Systolic blood pressure (mm Hg) ≤90, n (%)		1281 (13.4)	165 (13.2)	129 (3)	771 (4.8)	362 (1.8)	318 (6.1)	594 (3.1)	893 (18.7)	4513 (5.6)
Glasgow Coma Scale score, median (IQR)		15 (14-15)	14 (13-15)	15 (14-15)	15 (14-15)	15 (15-15)	14 (11-15)	15 (14-15)	3 (3-6)	15 (14-15)
Glasgow Coma Scale category										
	13-15, n (%)	8197 (87.8)	980 (79.7)	3829 (91.8)	14,659 (93.9)	19,028 (97.8)	3635 (70.8)	16,067 (85.2)	23 (0.5)	66,418 (84.6)
	9-12, n (%)	673 (7.2)	99 (8)	215 (5.2)	762 (4.9)	393 (2)	620 (12.1)	2122 (11.3)	96 (2)	4980 (6.3)
	3-8, n (%)	464 (5)	151 (12.3)	125 (3)	191 (1.2)	44 (0.2)	882 (17.2)	666 (3.5)	4593 (97.5)	7,116 (9.1)
	Missing, n (%)	222 (2.3)	23 (1.8)	139 (3.2)	448 (2.8)	961 (4.7)	99 (1.9)	503 (2.6)	55 (1.2)	2450 (3)
	Body temperature (℃), median (IQR)	36.5 (36-36.9)	36.4 (36- 36.8)	36.7 (36.3- 37)	36.6 (36.3-37)	36.7 (36.4-37)	36.4 (36- 36.8)	36.5 (36.1-36.8)	36.1 (35.5-36.5)	36.6 (36.2-36.9)
	Lactate on arrival (mmol/L), median (IQR)	2.33 (1.50-3.60)	2.23 (1.44- 3.70)	1.55 (1- 2.30)	2 (1.40- 2.90)	1.50 (1-2.20)	2.22 (1.50- 3.32)	1.72 (1.20- 2.59)	3 (1.80- 6)	1.90 (1.22- 2.90)
Injured body regions of AIS^b^ >2										
	Head and cervical, n (%)	1433 (15)	491 (39.2)	1046 (24.3)	2160 (13.4)	588 (2.9)	3311 (63.2)	16,298 (84.2)	4265 (89.5)	29,592 (36.5)
	Face, n (%)	0 (0)	13 (1)	1 (0)	0 (0)	0 (0)	269 (5.1)	0 (0)	5 (0.1)	288 (0.4)
	Chest, n (%)	5010 (52.4)	362 (28.9)	659 (15.3)	8850 (55.1)	798 (3.9)	1650 (31.5)	1018 (5.3)	1697 (35.6)	20,044 (24.8)
	Abdomen, n (%)	4597 (48.1)	89 (7.1)	101 (2.3)	0 (0)	15 (0.1)	240 (4.6)	53 (0.3)	198 (4.2)	5293 (6.5)
	Extremities, n (%)	2158 (22.6)	247 (19.7)	2360 (54.8)	4161 (25.9)	17,009 (83.3)	970 (18.5)	262 (1.4)	733 (15.4)	27,900 (34.5)
	External, n (%)	0 (0)	180 (14.4)	0 (0)	0 (0)	0 (0)	0 (0)	0 (0)	0 (0)	180 (0.2)
	Multiregional injuries with AIS >2, n (%)	3876 (40.6)	329 (26.3)	317 (7.4)	2554 (15.9)	519 (2.5)	1737 (33.2)	1207 (6.2)	1738 (36.5)	12,277 (15.2)
	RTS^c^, median (IQR)	7.84 (7.55- 7.84)	7.84 (7.11-7.84)	7.84 (7.84- 7.84)	7.84 (7.84- 7.84)	7.84 (7.84-7.84)	7.84 (6.90- 7.84)	7.84 (7.84- 7.84)	4.09 (4.09- 5.97)	7.84 (7.55- 7.84)
	ISS^d^, median (IQR)	17 (11-25)	17 (9- 24)	9 (9-14)	9 (9-16)	9 (9-9)	20 (13-29)	16 (9-17)	25 (18-33)	10 (9- 18)
	TRISS^e^ Ps, median (IQR)	0.96 (0.90- 0.99)	0.96 (0.89- 0.98)	0.97 (0.94- 0.97)	0.98 (0.96- 0.99)	0.97 (0.97- 0.97)	0.94 (0.85- 0.98)	0.94 (0.92- 0.98)	0.45 (0.26- 0.68)	0.97 (0.93- 0.98)
	TRISS Ps >0.5, n (%)	8994 (94.1)	1148 (91.6)	4228 (98.1)	15,898 (99.0)	20,378 (99.8)	4794 (91.6)	19,094 (98.6)	2115 (44.4)	76,649 (94.7)
	Transfusion within 24 h, n (%)	3067 (33.2)	338 (27.6)	485 (11.5)	1668 (10.8)	1704 (8.7)	1158 (22.9)	1197 (6.4)	1964 (43.2)	11,581 (14.9)
	Missing, n (%)	321 (3.4)	29 (2.3)	105 (2.4)	626 (3.9)	787 (3.9)	184 (3.5)	765 (4)	216 (4.5)	3033 (3.7)
	pRBC^f^ within 24 h, units, median (IQR)	6 (4-14)	4 (2-10)	2 (2-4)	4 (2-8)	2 (2-4)	6 (2-10)	2 (0-4)	6 (4-12)	4 (2-8)
	FFP^g^ within 24 h (units), median (IQR)	8 (4-16)	6 (4-12)	4 (0-6)	4 (2-10)	1 (0-6)	8 (4-14)	4 (0-8)	8 (4-16)	6 (2-12)
	PC^h^ within 24 h (units), median (IQR)	0 (0-20)	0 (0-10)	0 (0-8)	0 (0-4)	0 (0-0)	0 (0-10)	0 (0-1)	0 (0-15)	0 (0-10)
	Administration of TXA^i^, n (%)	2353 (24.6)	306 (24.4)	410 (9.5)	1845 (11.5)	769 (3.8)	1436 (27.4)	3355 (17.3)	1679 (35.2)	12,153 (15)
	Survivors, n (%)	9055 (94.8)	1170 (93.4)	4007 (93)	15,783 (98.3)	20,137 (98.6)	4930 (94.2)	18,606 (96.1)	2393 (50.2)	76,081 (94)

^a^Data are presented as median (IQR) for continuous variables and number (percentage) for categorical variables. For categorical variables containing missing values (Glasgow Coma Scale categories and 24-hour blood transfusion), percentages of the documented response categories were calculated using the nonmissing denominator, whereas the Missing row reports the proportion relative to the full cohort.

^b^AIS: Abbreviated Injury Scale.

^c^RTS: Revised Trauma Score.

^d^ISS: Injury Severity Score.

^e^TRISS Ps: Trauma and Injury Severity Score predicted survival probability.

^f^pRBC: packed red blood cells.

^g^FFP: fresh frozen plasma.

^h^PC: platelet concentrate.

^i^TXA: tranexamic acid.

Prior to applying the random forest imputation, the proportions of missing data were evaluated. Because blank AIS fields were assigned as “0” (no injury) based on registry specifications, the missing rate for all anatomical AIS variables was 0% in both cohorts. The preimputation missing rates for all variables are provided in Table S2 in Multimedia Appendix 1.

### Development of the Multivariable Integrated Model

In the derivation cohort (2015-2018), multivariable logistic regression analysis showed that belonging to trauma phenotype 8 was a risk factor for mortality after adjusting for baseline TRISS predictions (odds ratio 2.38, 95% CI 2.11-2.68; *P*<.001; Table S3 in Multimedia Appendix 1). This approach preserved probabilistic interpretability. The variance inflation factor values for the logit-transformed TRISS probability and phenotype categories were both 1.65, confirming no multicollinearity.

### Temporal Validation of the Integrated Model

In the temporal validation cohort (2019-2022), the baseline TRISS model yielded an AUROC of 0.889 (95% CI 0.884-0.894). Calibration plots of the baseline TRISS model across trauma phenotypes (Figure S7 in Multimedia Appendix 1), age categories (Figure S8 in Multimedia Appendix 1), and severity of head and cervical injury (Figure S9 in Multimedia Appendix 1) are provided in Multimedia Appendix 1. The integrated multivariable model yielded higher performance metrics than the baseline TRISS model (Table 2).

**Table 2:** Predictive performance of the baseline Trauma and Injury Severity Score model and the integrated multivariable model in the temporal validation cohort (Japan Trauma Data Bank 2019-2022).

Performance metric			Baseline TRISS^a^		Integrated model
Overall discrimination					
	AUROC^b^ (95% CI)	0.889 (0.884-0.894)		0.897 (0.892-0.902)^c^	
Classification accuracy (threshold=0.5)					
	Sensitivity	0.472		0.341	
	Specificity	0.974		0.988	
Overall performance and calibration					
	Brier score	0.0454		0.0394	
	Logarithmic loss (LogLoss)	0.1670		0.1458	
Reclassification					
	Continuous NRI^d^	Reference		0.0123	
	IDI^e^	Reference		−0.0552	

^a^TRISS: Trauma and Injury Severity Score.

^b^AUROC: area under the receiver operating characteristic curve.

^c^*P*<.001 compared to the baseline TRISS model (DeLong test).

^d^NRI: net reclassification improvement.

^e^IDI: integrated discrimination improvement.

**Figure 2:**
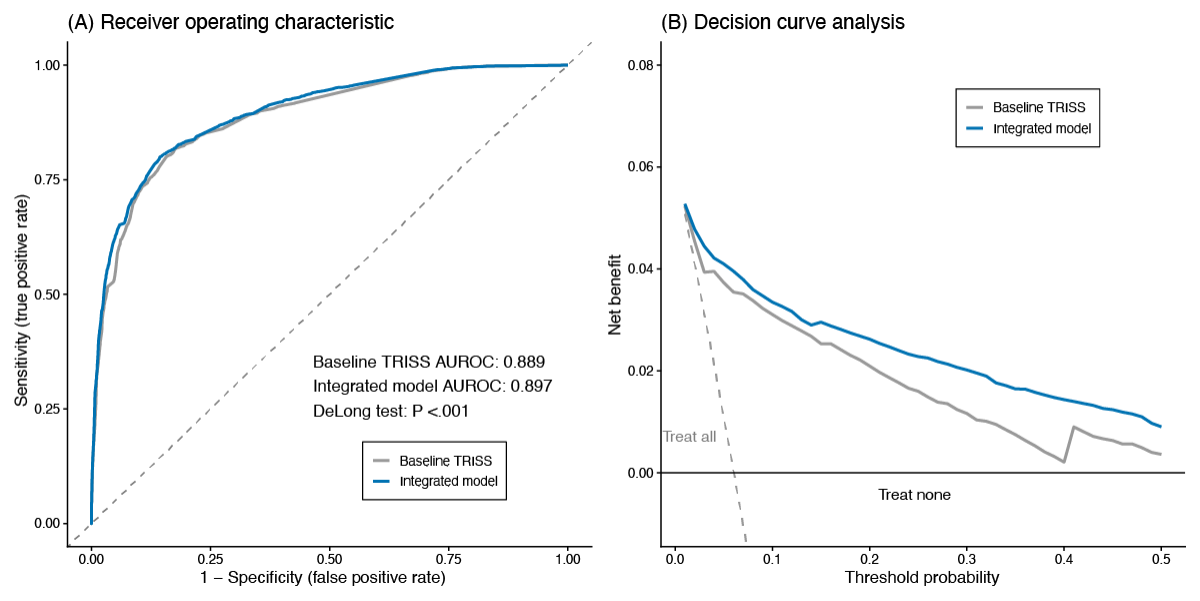
Predictive performance of the integrated multivariable model and the baseline TRISS model in the temporal validation cohort (JTDB 2019-2022, n=80,964). (A) Receiver operating characteristic (ROC) curve showing significantly improved discrimination (AUROC: 0.897 vs 0.889; DeLong test *P*<.001). (B) Decision curve analysis (DCA) demonstrates the comparative net benefit across clinically relevant threshold probabilities. The integrated model provides a consistently higher net benefit compared to the standard TRISS model. AUROC: area under the receiver operating characteristic; TRISS: Trauma and Injury Severity Score.

The integrated model achieved an AUROC of 0.897 (95% CI 0.892-0.902; DeLong test *P*<.001; Figure 2A).

Using a predicted mortality probability threshold of 0.5, the baseline TRISS model demonstrated a sensitivity of 0.472 and specificity of 0.974. In comparison, the integrated multivariable model achieved a sensitivity of 0.341 and specificity of 0.988. The Brier Score was 0.0394, and the LogLoss was 0.1458. The continuous net reclassification improvement was 0.0123, and the IDI was −0.0552.

To provide an assessment of model calibration, we calculated the calibration metrics. In the overall validation cohort, the integrated model showed a calibration intercept of –0.152, slope of 0.965, and Brier score of 0.0394. Side-by-side calibration plots for trauma phenotype 8 were generated for both cohorts (Figure S10 in Multimedia Appendix 1). These plots show that multivariable integration mitigates the underestimation of mortality observed in patients with trauma phenotype 8. Quantitative calibration metrics for each of the 8 clusters are summarized in Table S4 in Multimedia Appendix 1.

The clinical utility of the integrated model was assessed using DCA in the validation cohort. The DCA plot showed that the multivariable integrated model provided a higher Net Benefit than the standard TRISS model across the evaluated threshold probabilities (Figure 2B).

### Implementation of the Online Platform

**Figure 3:**
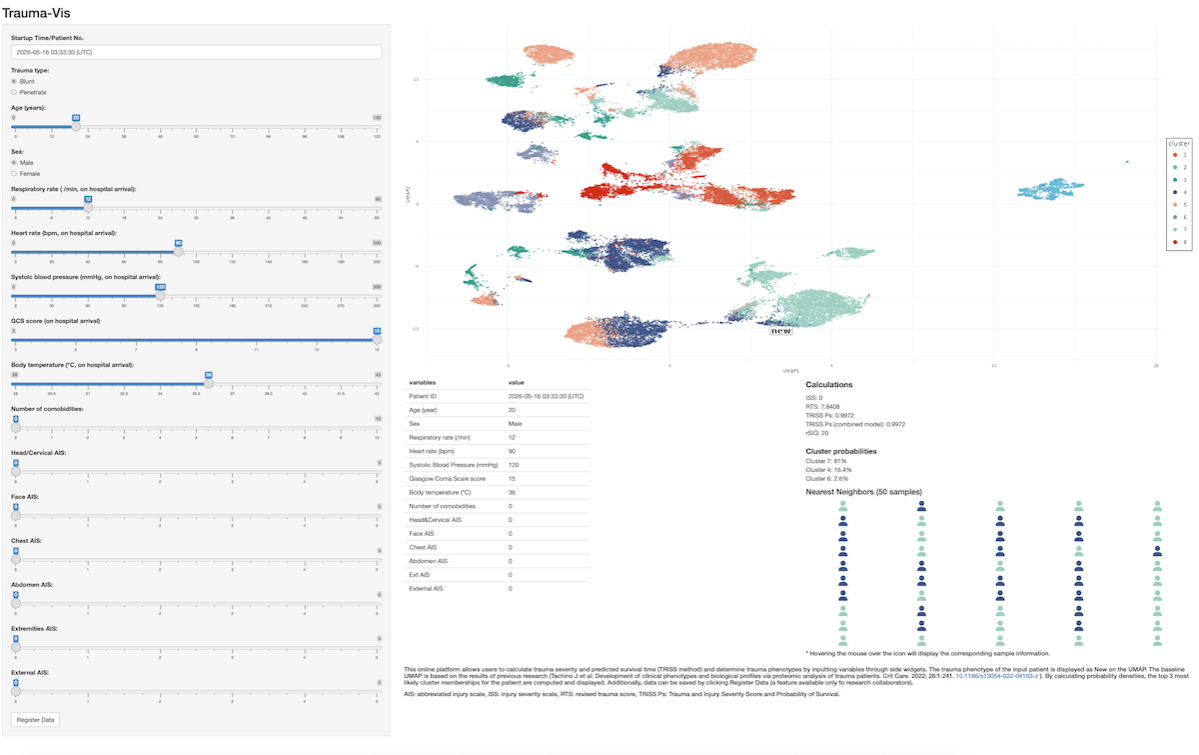
User interface of the online platform (Trauma-Vis). Screenshot of the online platform interface showing the trauma phenotype identification system. The left panel allows users to input 14 routinely collected clinical variables via slide widgets. The upper right panel displays the inputted patient’s assigned trauma phenotype as a star on the uniform manifold approximation and projection (UMAP) visualization. The lower right panel provides real-time calculations of conventional trauma scores (eg, TRISS, RTS, and ISS), cluster probabilities, and characteristics of the 50 nearest neighbors. AIS: Abbreviated Injury Scale; GCS: Glasgow Coma Scale; ISS: Injury Severity Score; RTS: Revised Trauma Score; TRISS: Trauma and Injury Severity Score.

The platform provides trauma metrics and phenotype-based risk stratification within a single interface (Figure 3).

The 14 input variables required by Trauma-Vis are routinely collected during standard trauma surveys upon arrival in the emergency department. A simulation indicated that data entry into the web interface can be completed in approximately 1 minute, providing phenotype classification and mortality prediction.

## Discussion

### Principal Findings

This study addresses 2 challenges in modern trauma care: the limitations of the conventional TRISS model’s predictive accuracy and the translation of machine learning prognostication into a practical clinical tool. First, by applying a multivariable logistic regression framework, we observed that integrating machine learning–derived trauma phenotypes improved prognostic performance. This integration mitigated the underestimation of mortality previously observed in high-risk subgroups, leading to improvements in overall discrimination, calibration, and clinical utility based on raw mortality outcomes. Furthermore, DCA demonstrated a higher net benefit for the integrated model across clinically relevant threshold probabilities. Second, we developed “Trauma-Vis” as a freely accessible web-based platform to provide phenotype assessments to clinicians.

### Comparison With Prior Work

Our analysis evaluated TRISS model performance using data from a national trauma registry. The observed baseline TRISS AUROC of 0.889 is consistent with previous findings [11,12,20]. Additionally, the underestimation of survival in low-probability cases is consistent with findings from the National Trauma Data Bank [18]. An age-stratified analysis revealed that calibration was closest to the ideal line in patients aged ≥81 years (Figure S8 in Multimedia Appendix 1), potentially because the model’s tendency to underestimate survival is counterbalanced by age-related mortality risk [36]. A recent meta-analysis of trauma scoring systems in older patients reported an AUROC of 0.82 [37]. Furthermore, the historical misclassification of mortality risk among patients with severe traumatic brain injury (TBI; Figure S9 in Multimedia Appendix 1) suggests a limited capacity of the baseline model to fully capture the pathophysiology of neurological trauma.

By integrating trauma phenotypes, our multivariable model recalibrated these predictions. This recalibration also explains the shift observed in classification metrics. Because the baseline TRISS model tended to overestimate the mortality risk, correcting this overestimation shifted the overall predicted probabilities downward toward the true low prevalence (6%). Consequently, when evaluated at a fixed 0.5 threshold, the integrated model yielded a lower sensitivity (0.341 vs 0.472) but a higher specificity (0.988 vs. 0.974), thereby reducing false-positive mortality predictions for actual survivors. This phenomenon suggests the limitation of relying on a single probability cutoff in imbalanced datasets and supports the evaluation of models using continuous metrics and DCA. Similarly, the negative IDI (−0.0552) indicates a slight narrowing of the discrimination slope, which is consistent with this calibration correction; because IDI is based on absolute differences in mean predicted probabilities, whereas AUROC reflects rank ordering, these metrics can move in different directions when an overestimating model is recalibrated. This phenotype-based approach aligns with prognostic enrichment strategies in precision medicine [21]. With the anticipated advancement of individualized diagnosis for critically ill patients and improved enrichment in clinical trials [38,39], trauma phenotype identification could facilitate the implementation of prognostic enrichment. Operationally, the trauma phenotype model uses the same 14 input variables as the conventional TRISS. This compatibility allows for integration into existing trauma assessment protocols without requiring additional data collection, which is a practical consideration given that TRISS remains a widely used outcome prediction tool.

### Clinical Plausibility

The observed improvement in predictive performance upon integrating machine learning–derived phenotypes with the TRISS model may be explained by the ability of phenotyping to classify highly heterogeneous pathophysiological states from a perspective distinct from that of conventional prediction models. Traditional scoring systems typically assess overall severity using predefined combinations of variables, whereas computational phenotyping identifies multidimensional, data-driven patterns of patient states. This complementary concept is supported by recent studies in other critical care domains. For instance, in patients with sepsis, identifying data-driven clinical phenotypes has allowed for the classification of heterogeneous host-response patterns that are not fully captured by traditional severity scores [40]. Furthermore, in TBI, the addition of unsupervised clustering endotypes to the established International Mission for Prognosis and Analysis of Clinical trials in TBI model was found to have improved its prognostic precision [41]. Consistent with these reports, our findings suggest that incorporating comprehensive phenotype membership provides additional pathophysiological context to the conventional TRISS model. Because multiple phenotypes (eg, phenotypes 3, 5, 6, 7, and 8) exhibited significant independent associations with mortality, this multidimensional classification refines mortality prediction, correcting both overestimation and underestimation, without requiring the collection of new clinical variables.

### Actionable Clinical Interventions

A patient’s specific trauma phenotype can inform tailored clinical interventions at the bedside. Rather than relying solely on a single overall severity score, our integrated model leverages the distinct pathophysiological profiles of multiple phenotypes to guide precise management. For instance, phenotype 8, characterized by a mortality rate of approximately 50%, constitutes a high-risk subgroup that can be underrecognized by traditional scoring systems. A previous proteomic analysis indicating systemic inflammation and early acute traumatic coagulopathy in phenotype 8 provides a biological rationale for targeted interventions [23]. Therefore, Trauma-Vis has the potential to function as a clinical decision support tool. When the platform flags a patient as phenotype 8 during the “golden hour” [42], it could serve as an early indicator of clinical deterioration. This awareness may prompt clinicians to consider preemptively implementing interventions, such as the activation of massive transfusion protocols, administration of coagulation factors, or damage control procedures, ahead of standard physiological triggers, even if the baseline TRISS score indicates a high probability of survival [24]. Conversely, identifying phenotypes with significantly distinct risk profiles (eg, phenotypes 3, 5, 6, or 7) could guide alternative resuscitation strategies or help clinicians avoid over-triage by recognizing when a patient’s actual physiological state is more robust than predicted by conventional baseline scores.

### Limitations

This study has several limitations. First, our assessment used temporally distinct derivation and validation cohorts drawn from the same national database (JTDB). This split-sample approach constitutes an internal, temporal validation, not a true external validation [10]. Differences in trauma epidemiology, prehospital transport systems, and ICU practices across countries could alter the phenotype distribution and model calibration. Furthermore, as trauma care advances (eg, evolving transfusion strategies and shifts between surgical and endovascular management), temporal changes may introduce concept drift into the fixed phenotyping algorithm. Thus, calibration should be continually monitored, and the clustering model may require recalibration when applied to future cohorts. However, clinical deployment should follow geographic external validation across health care systems. Second, the retrospective design inherently carries the risks of residual confounding. Third, using the TRISS for prospective, patient-level prediction departs from its original role in retrospective, system-level performance assessment; this shift necessitates caution in individual-level clinical prognostication.

### Future Directions

Although this study used a forward-facing temporal validation, the analysis was fundamentally retrospective. Therefore, true prospective validation studies within domestic trauma centers are essential to confirm the robustness and real-time clinical utility of the model. Furthermore, multicenter geographic external validation across international health care systems should be pursued to evaluate the transportability of the integrated model. Prospective usability studies are also needed to evaluate manual data entry time, clinician interpretation of visualizations, and the impact of real-time phenotyping on clinical workflows during initial trauma care. Regarding workflow integration, a preliminary simulated test indicated that manual data entry of the 14 routinely collected variables into the web interface can be completed in approximately 1 minute.

### Conclusions

Integrating machine learning–derived trauma phenotypes with the TRISS via multivariable modeling improved the accuracy, calibration, and clinical net benefit of in-hospital mortality prediction compared to the baseline model. The developed online platform demonstrated the technical feasibility of real-time trauma phenotype identification, providing a potential pathway for decision support at the bedside.

## Data Availability

The underlying code for implementing the proprietary phenotype classification and related decision logic is not publicly available due to an ongoing international patent application. Nonproprietary analysis scripts (eg, data wrangling, statistical analyses, and figure generation) and an explanatory description of the algorithmic workflow can be provided to qualified researchers upon reasonable request to the corresponding author, subject to institutional approval and appropriate nondisclosure and data-use agreements. The training and validation datasets and any parameter files that could disclose protected implementation details are not publicly available for the same reason. The datasets generated and/or analyzed during the current study are not publicly available due to data-use agreements with the Japan Trauma Data Bank. The web-based platform is freely accessible for academic use [43].

## Appendix

Multimedia Appendix 1 Additional methods, tables, and figures for trauma phenotype assignment, patient selection, baseline characteristics, missing data, and model calibration in the derivation and validation cohorts.

Multimedia Appendix 2 Graphical Abstract.

## Abbreviations

AIS: Abbreviated Injury Scale
AUROC: area under the receiver operating characteristic
DCA: decision curve analysis
ICU: intensive care unit
IDI: integrated discrimination improvement
ISS: Injury Severity Score
JTDB: Japan Trauma Data Bank
rSIG: reverse shock index multiplied by the Glasgow Coma Scale score
RTS: Revised Trauma Score
STROBE: Strengthening the Reporting of Observational Studies in Epidemiology
TBI: traumatic brain injury
TRIPOD: Transparent Reporting of a multivariable prediction model for Individual Prognosis or Diagnosis
TRISS: Trauma and Injury Severity Score
