# Supplementary material for: Novel Online Platform for Trauma Care—Integrating Trauma Phenotypes to Optimize the Trauma and Injury Severity Score Model: Retrospective Cohort Study
Source: JMIR Med Inform. 2026 Jun 2;14:e90011. doi: 10.2196/90011 (PMC13273197; doi:10.2196/90011)
Supplement: Multimedia Appendix 1 [file medinform_v14i1e90011_app1.docx]

**Supplemental Digital Content**

**Novel online platform for trauma care: Integrating trauma phenotypes to optimize the Trauma and Injury Severity Score model: Retrospective Cohort Study**

***Database***

The Japan Trauma Data Bank (JTDB), established in 2003, is a comprehensive trauma registry that encompasses data from 303 hospitals as of March 2022. Initiated by the Japanese Association for the Surgery of Trauma (Trauma Registry Committee) and the Japanese Association for Acute Medicine (Committee for Clinical Care Evaluation), the JTDB aims to enhance the quality of trauma care in Japan. The data, updated continuously, were shared online and stored on the data server of the Association for Japan Trauma Care and Research. The JTDB primarily records patients from tertiary care and emergency centers suspected of sustaining injuries with an Abbreviated Injury Scale score of 3 or higher, and tracks their progress until hospital discharge or death.

***Imputation of Missing Values***

To handle missing data without introducing anatomical confounding, we employed a two-step approach. First, consistent with standard trauma registry practices where blank fields indicate the absence of injury, missing AIS values for all six body regions were deterministically coded as "0" (no injury) prior to applying any imputation algorithms. As a result, the pre-imputation missing rate for all anatomical AIS variables was exactly 0.00% (Supplemental Table 2).

Subsequently, missing values exclusively for physiological and demographic variables were imputed using machine learning-based imputation techniques with the "missForest" package. This technique employs a nonparametric algorithm capable of capturing nonlinear relationships and interactions between variables using random forests to generate estimated values [1, 2]. The advantages of using random forest models include their ability to handle both categorical and continuous variables, minimal tuning requirements, and robust internal validation through out-of-bag error estimation. This imputation method has been reported to demonstrate superior reliability and effectiveness compared to other approaches, such as k-nearest neighbors and multivariate imputation (including chained equation models).

***Trauma-Vis***

A workflow diagram of the phenotype assignment process and multivariable probability calculation pipeline is provided in Supplemental Figure 1. The platform calculates conventional trauma outcome prediction models (ISS, RTS, TRISS, and rSIG) while visualizing the trauma phenotype. The platform is freely available for academic use at <https://github.com/jotarotachino/Trauma-Vis>.

***References***

1. Stekhoven DJ, Buhlmann P (2012) MissForest--non-parametric missing value imputation for mixed-type data. Bioinformatics 28:112-118 <https://doi.org/10.1093/bioinformatics/btr597>

2. Waljee AK, Mukherjee A, Singal AG, Zhang Y, Warren J, Balis U, Marrero J, Zhu J, Higgins PD (2013) Comparison of imputation methods for missing laboratory data in medicine. BMJ Open 3:e002847 <https://doi.org/10.1136/bmjopen-2013-002847>

**Table S1.** Baseline characteristics in the derivation cohort (JTDB 2015–2018).

|  | Trauma phenotype 1 | Trauma phenotype 2 | Trauma phenotype 3 | Trauma phenotype 4 | Trauma phenotype 5 | Trauma phenotype 6 | Trauma phenotype 7 | Trauma phenotype 8 | Overall |
| --- | --- | --- | --- | --- | --- | --- | --- | --- | --- |
|  | N=9,919 | N=4,477 | N=6,460 | N=17,384 | N=17,377 | N=6,125 | N=20,958 | N=5,182 | N=87,882 |
| Age, years, median [IQR] | 51 [31, 68] | 49 [27, 69] | 81 [74, 87] | 41 [22, 59] | 78 [66, 86] | 51 [29, 70] | 67 [49, 78] | 68 [49, 79] | 65 [41, 79] |
| Male sex, No. (%) | 6,873 (69.3) | 3,133 (70.0) | 2,594 (40.2) | 14,169 (81.5) | 5,059 (29.1) | 4,450 (72.7) | 15,018 (71.7) | 3,518 (67.9) | 54,814 (62.4) |
| Number of comorbidities, median [IQR] | 0 [0, 1] | 0 [0, 1] | 3 [3, 4] | 0 [0, 1] | 1 [0, 2] | 0 [0, 1] | 1 [0, 1] | 0 [0, 1] | 1 [0, 2] |
| Respiratory rate (/min), median [IQR] | 22 [18, 26] | 20 [18, 24] | 19 [17, 22] | 21 [18, 25] | 19 [16, 21] | 20 [18, 24] | 19 [16, 22] | 20 [16, 24] | 20 [17, 24] |
| Heart rate (bpm), median [IQR] | 87 [74, 102] | 85 [74, 97] | 81 [71, 93] | 86 [75, 100] | 80 [70, 90] | 86 [74, 100] | 80 [70, 91] | 90 [73, 110] | 83 [72, 96] |
| Systolic blood pressure (mmHg), median [IQR] | 125 [105, 143] | 134 [119, 154] | 151 [131, 170] | 130 [116, 147] | 149 [129, 168] | 136 [118, 156] | 144 [124, 165] | 138 [102, 169] | 139 [120, 160] |
| Systolic blood pressure (mmHg) ≤ 90, No. (%) | 1,350 (13.6) | 239 (5.3) | 153 (2.4) | 805 (4.6) | 367 (2.1) | 381 (6.2) | 694 (3.3) | 1,040 (20.1) | 5,029 (5.7) |
| Glasgow Coma Scale score, median [IQR] | 15 [14, 15] | 15 [14, 15] | 15 [14, 15] | 15 [14, 15] | 15 [15, 15] | 14 [12, 15] | 14 [13, 15] | 3 [3, 6] | 15 [14, 15] |
| *Glasgow Coma Scale category* |  |  |  |  |  |  |  |  |  |
| 13–15, No. (%) | 8,592 (86.6) | 3,829 (85.5) | 6,035 (93.4) | 16,273 (93.6) | 17,014 (97.9) | 4,393 (71.7) | 17,723 (84.6) | 24 (0.5) | 73,883 (84.1) |
| 9–12, No. (%) | 781 (7.9) | 268 (6.0) | 300 (4.6) | 885 (5.1) | 311 (1.8) | 742 (12.1) | 2,436 (11.6) | 91 (1.8) | 5,814 (6.6) |
| 3–8, No. (%) | 546 (5.5) | 380 (8.5) | 125 (1.9) | 226 (1.3) | 52 (0.3) | 990 (16.2) | 799 (3.8) | 5,067 (97.8) | 8,185 (9.3) |
| Body temperature (℃), median [IQR] | 36.4 [36.0, 36.8] | 36.5 [36.1, 36.9] | 36.6 [36.3, 37.0] | 36.6 [36.2, 37.0] | 36.6 [36.3, 37.0] | 36.4 [36.0, 36.8] | 36.4 [36.0, 36.8] | 36.0 [35.4, 36.5] | 36.5 [36.1, 36.9] |
| *Injured body regions of AIS >2* |  |  |  |  |  |  |  |  |  |
| Head & Cervical, No. (%) | 1,463 (14.7) | 1,431 (32.0) | 1,563 (24.2) | 2,364 (13.6) | 755 (4.3) | 3,681 (60.1) | 17,571 (83.8) | 4,683 (90.4) | 33,511 (38.1) |
| Face, No. (%) | 3 (0.0) | 37 (0.8) | 1 (0.0) | 0 (0.0) | 0 (0.0) | 649 (10.6) | 0 (0.0) | 14 (0.3) | 704 (0.8) |
| Chest, No. (%) | 4,938 (49.8) | 1,059 (23.7) | 815 (12.6) | 8,391 (48.3) | 643 (3.7) | 1,604 (26.2) | 826 (3.9) | 1,947 (37.6) | 20,223 (23.0) |
| Abdomen, No. (%) | 4,369 (44.0) | 241 (5.4) | 81 (1.3) | 0 (0.0) | 23 (0.1) | 213 (3.5) | 74 (0.4) | 212 (4.1) | 5,213 (5.9) |
| Extremities, No. (%) | 2,460 (24.8) | 825 (18.4) | 3,575 (55.3) | 5,206 (29.9) | 13,546 (78.0) | 1,233 (20.1) | 353 (1.7) | 883 (17.0) | 28,081 (32.0) |
| External, No. (%) | 0 (0.0) | 56 (1.3) | 0 (0.0) | 0 (0.0) | 0 (0.0) | 0 (0.0) | 0 (0.0) | 0 (0.0) | 56 (0.1) |
| Multi-regional injuries with AIS> 2, No. (%) | 3,896 (39.3) | 651 (14.5) | 375 (5.8) | 2,709 (15.6) | 607 (3.5) | 1,887 (30.8) | 1,143 (5.5) | 2,057 (39.7) | 13,325 (15.2) |
| RTS, median [IQR] | 7.84 [7.55, 7.84] | 7.84 [7.84, 7.84] | 7.84 [7.84, 7.84] | 7.84 [7.84, 7.84] | 7.84 [7.84, 7.84] | 7.84 [6.90, 7.84] | 7.84 [7.84, 7.84] | 4.09 [4.09, 5.97] | 7.84 [7.84, 7.84] |
| ISS, median [IQR] | 18 [11, 29] | 10 [5, 19] | 9 [9, 14] | 10 [9, 17] | 9 [9, 9] | 20 [13, 29] | 16 [9, 17] | 25 [21, 34] | 10 [9, 20] |
| TRISS Ps, median [IQR] | 0.96 [0.89, 0.99] | 0.98 [0.94, 0.99] | 0.97 [0.94, 0.97] | 0.99 [0.96, 0.99] | 0.97 [0.97, 0.97] | 0.95 [0.88, 0.99] | 0.96 [0.93, 0.98] | 0.45 [0.21, 0.67] | 0.97 [0.93, 0.99] |
| TRISS Ps>0.5, No. (%) | 9,216 (92.9) | 4,291 (95.8) | 6,403 (99.1) | 17,201 (98.9) | 17,336 (99.8) | 5,681 (92.8) | 20,894 (99.7) | 2,178 (42.0) | 83,200 (94.7) |
| Transfusion within 24 h, No. (%) | 2,945 (29.7) | 493 (11.0) | 565 (8.7) | 1,427 (8.2) | 1,345 (7.7) | 994 (16.2) | 1,030 (4.9) | 1,931 (37.3) | 10,730 (12.2) |
| Missing, No. (%) | 1 (0.0) | 0 (0.0) | 2 (0.0) | 3 (0.0) | 2 (0.0) | 0 (0.0) | 4 (0.0) | 0 (0.0) | 12 (0.0) |
| Survivors, No. (%) | 9,275 (93.5) | 4,282 (95.6) | 6,199 (96.0) | 17,062 (98.1) | 17,148 (98.7) | 5,794 (94.6) | 20,127 (96.0) | 2,554 (49.3) | 82,441 (93.8) |

Data are presented as median [interquartile range] for continuous variables and number (percentage) for categorical variables. Variables in this table are post-imputation values. Pre-imputation missing rates are reported in Supplemental Table 2. For categorical variables containing missing values (24-hour blood transfusion), percentages of the documented response categories were calculated using the non-missing denominator, whereas the Missing row reports the proportion relative to the full cohort. AIS, Abbreviated Injury Scale; BT, body temperature; GCS, Glasgow Coma Scale; SBP, systolic blood pressure; HR, heart rate; RR, respiratory rate; ISS, Injury Severity Score; TRISS Ps, Trauma and Injury Severity Score predicted survival probability.

**Table S2.** Exact proportions of missing data for all variables prior to imputation in both cohorts.

| Variable | Missing percent Derivation (2015–2018) | Missing percent Validation (2019–2022) |
| --- | --- | --- |
| Age | 0 | 0 |
| Sex | 0 | 0 |
| Systolic blood pressure | 1.69 | 1.4 |
| Respiratory rate | 8.95 | 5.86 |
| Heart rate | 2.66 | 1.86 |
| Body temperature | 9.07 | 5.57 |
| Glasgow Coma Scale score | 5.84 | 3.03 |
| Number of comorbidities | 0 | 0.1 |
| Head & Cervical AIS | 0 | 0 |
| Face AIS | 0 | 0 |
| Chest AIS | 0 | 0 |
| Abdomen AIS | 0 | 0 |
| Extremities AIS | 0 | 0 |
| External AIS | 0 | 0 |

**Table S3.** Multivariable logistic regression model predicting in-hospital death in the derivation cohort (JTDB 2015–2018).

|  | Odds Ratio | 95% Confidence Interval | p-value |
| --- | --- | --- | --- |
| TRISS predicted mortality (logit transformed) | 2.38 | 2.33–2.44 | <0.001 |
|  |  |  |  |
| Trauma phenotypes |  |  |  |
| phenotype 1 | 1.00 (Reference) | – | – |
| phenotype 2 | 0.97 | 0.80–1.18 | 0.789 |
| phenotype 3 | 1.47 | 1.25–1.73 | <0.001 |
| phenotype 4 | 0.86 | 0.74–1.01 | 0.059 |
| phenotype 5 | 0.69 | 0.58–0.82 | <0.001 |
| phenotype 6 | 0.70 | 0.59–0.82 | <0.001 |
| phenotype 7 | 1.40 | 1.23–1.58 | <0.001 |
| phenotype 8 | 2.38 | 2.11–2.68 | <0.001 |

**Table S4.** Quantitative calibration metrics for the integrated multivariable model across the overall cohorts and individual trauma phenotypes.

| Cohort | Trauma phenotype | N | Intercept | Slope | Brier | AUC |
| --- | --- | --- | --- | --- | --- | --- |
| Derivation (2015–2018) | Overall | 87,882 | 0 | 1 | 0.0379 | 0.9163 |
|  | phenotype 1 | 9,919 | -0.1067 | 0.9437 | 0.0454 | 0.8867 |
|  | phenotype 2 | 4,477 | 0.2862 | 1.1399 | 0.0275 | 0.9373 |
|  | phenotype 3 | 6,460 | 0.3764 | 1.1331 | 0.0347 | 0.7213 |
|  | phenotype 4 | 17,384 | 0.5487 | 1.1862 | 0.0158 | 0.9072 |
|  | phenotype 5 | 17,377 | 0.8123 | 1.2033 | 0.0123 | 0.6893 |
|  | phenotype 6 | 6,125 | 0.1541 | 1.0786 | 0.0376 | 0.9161 |
|  | phenotype 7 | 20,958 | 0.8711 | 1.3237 | 0.035 | 0.8147 |
|  | phenotype 8 | 5,182 | -0.0056 | 0.6815 | 0.2083 | 0.7442 |
| Validation (2019–2022) | Overall | 80,964 | -0.1524 | 0.9648 | 0.0394 | 0.8972 |
|  | phenotype 1 | 9,556 | -0.3263 | 0.9406 | 0.0389 | 0.8803 |
|  | phenotype 2 | 1,253 | -0.2945 | 0.9521 | 0.0455 | 0.9059 |
|  | phenotype 3 | 4,308 | -0.1501 | 0.8008 | 0.0603 | 0.721 |
|  | phenotype 4 | 16,060 | -0.0031 | 1.0605 | 0.0156 | 0.8709 |
|  | phenotype 5 | 20,426 | -1.9118 | 0.5603 | 0.0144 | 0.6354 |
|  | phenotype 6 | 5,236 | -0.0155 | 1.0004 | 0.0433 | 0.8909 |
|  | phenotype 7 | 19,358 | -0.2585 | 0.9902 | 0.0359 | 0.7908 |
|  | phenotype 8 | 4,767 | 0.0283 | 0.636 | 0.2181 | 0.7203 |

**Supplemental Figures**

**Figure S1.** Workflow diagram detailing the trauma phenotype assignment process and multivariable probability calculation pipeline.

**
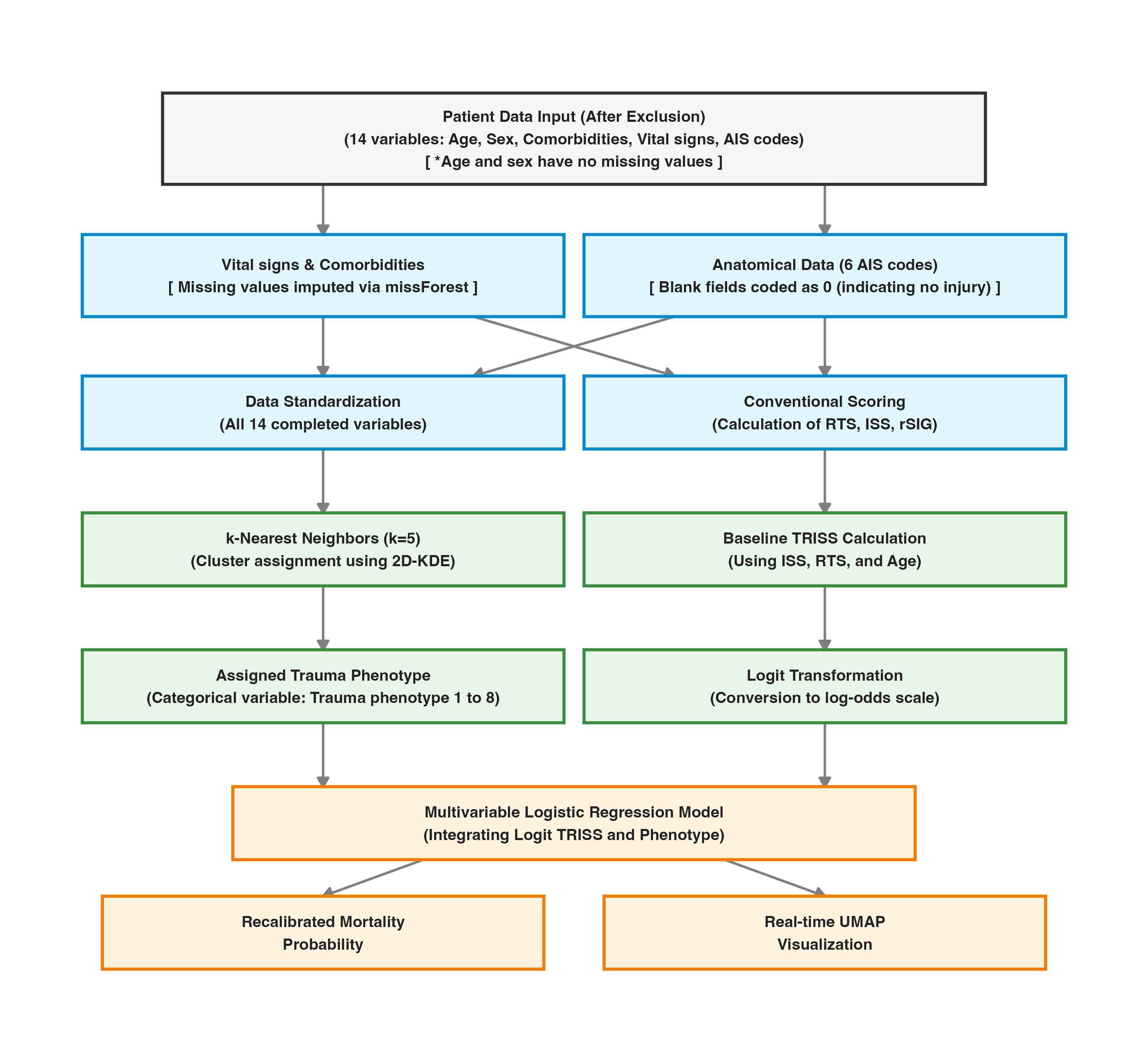
**

**Figure S2.** Patient selection flowchart for the historical derivation cohort.

**
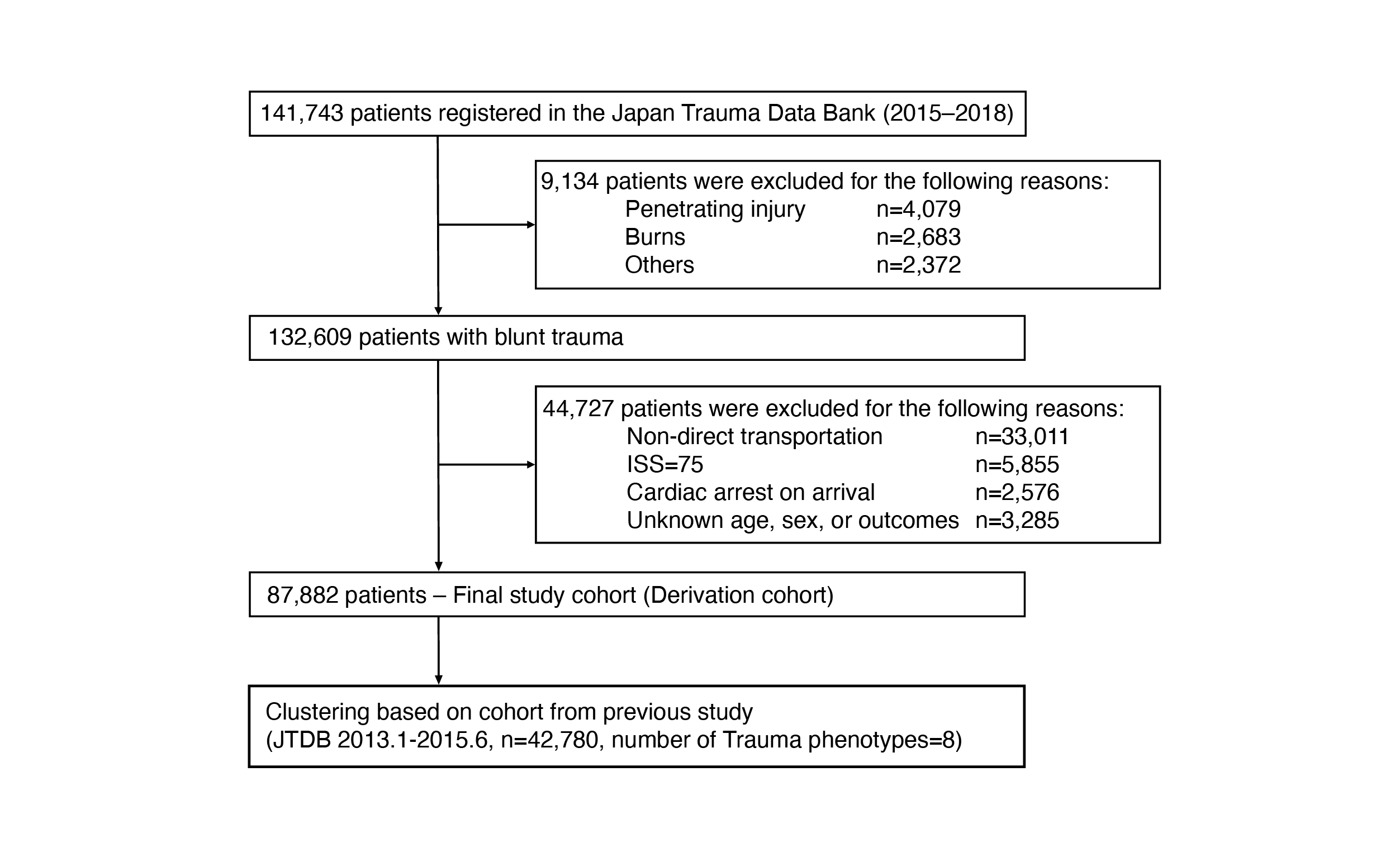
**

**
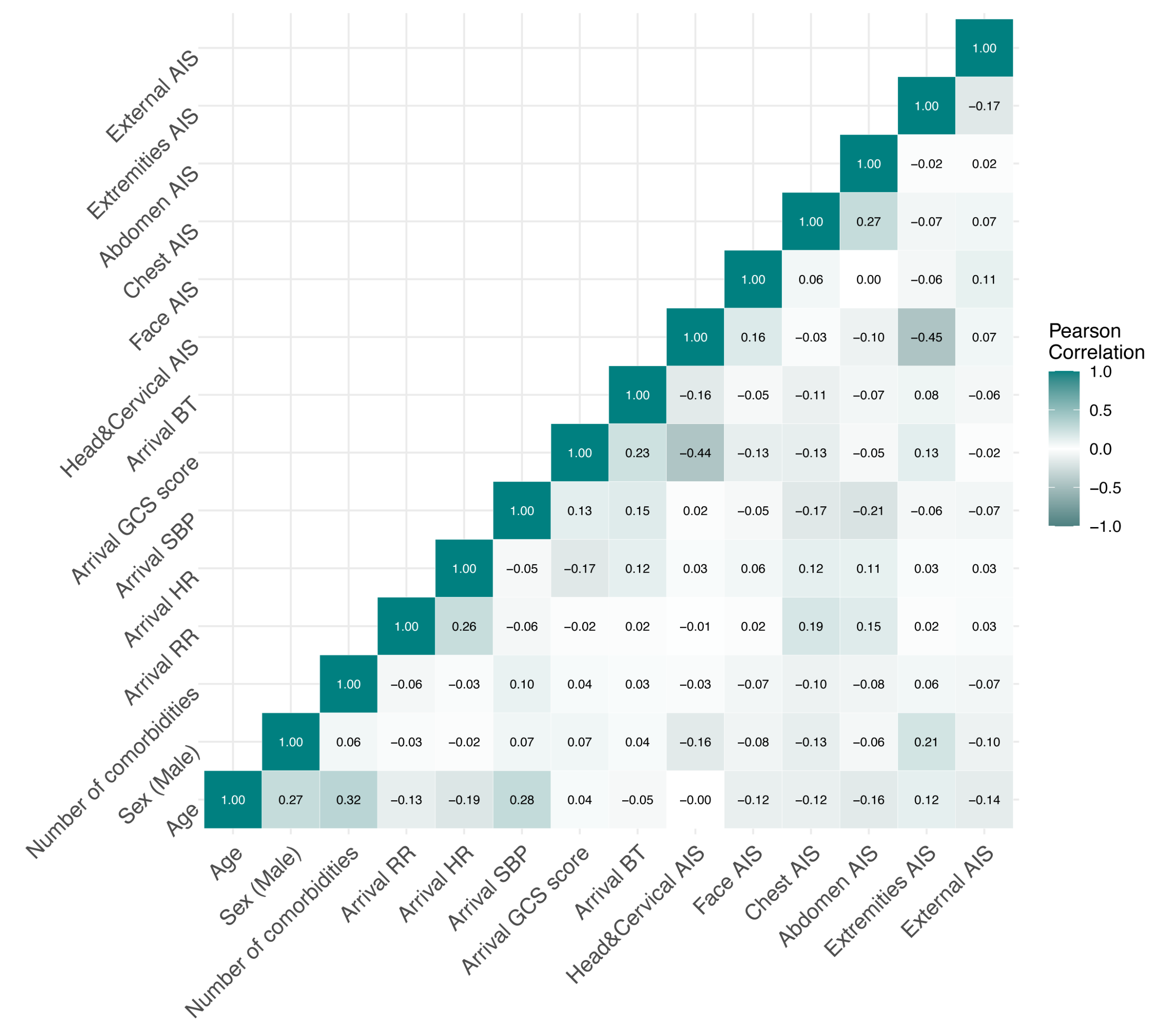
 Figure S3.** Correlation matrix heatmap showing the relationships between variables used for phenotype clustering.

**Figure S4.** Complex heatmap with the distribution of survival rates and variables for each trauma phenotype in the derivation cohort.

**
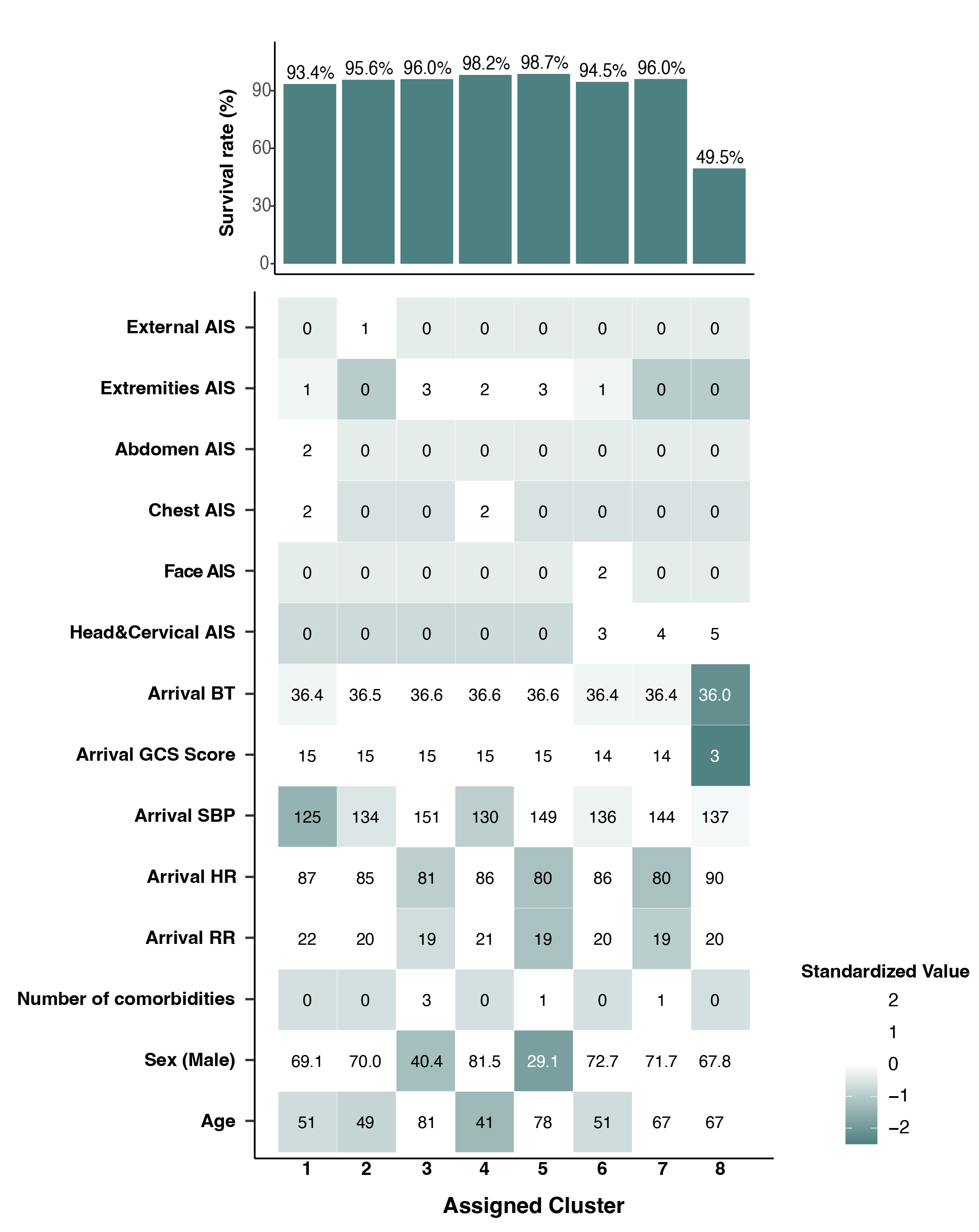
**

**Figure S5.** Calibration plots of the TRISS model for each trauma phenotype in the derivation cohort (JTDB 2015–2018)
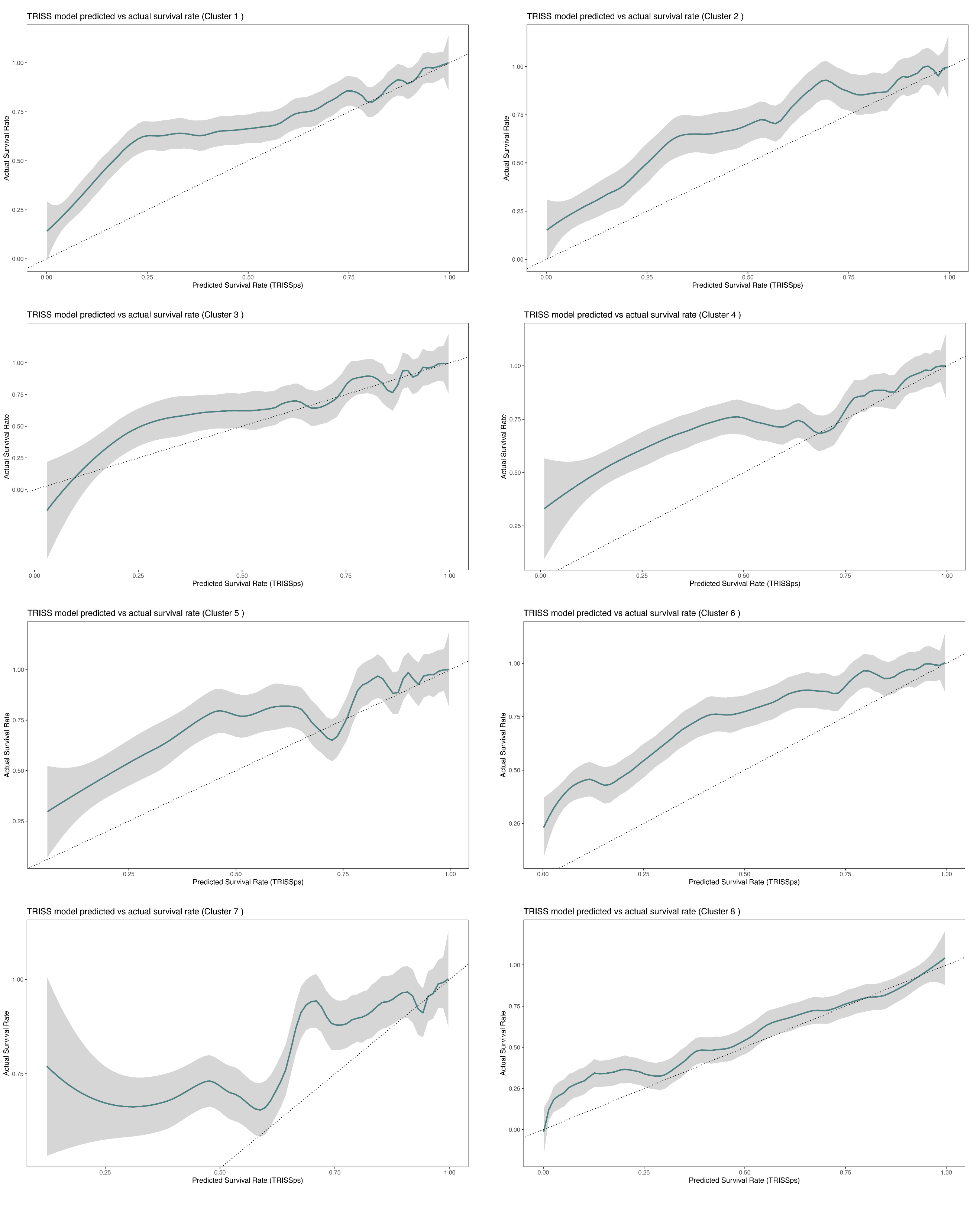
.

**Figure S6.** Complex heatmap with the distribution of survival rates and variables for each trauma phenotype in the validation cohort.

**
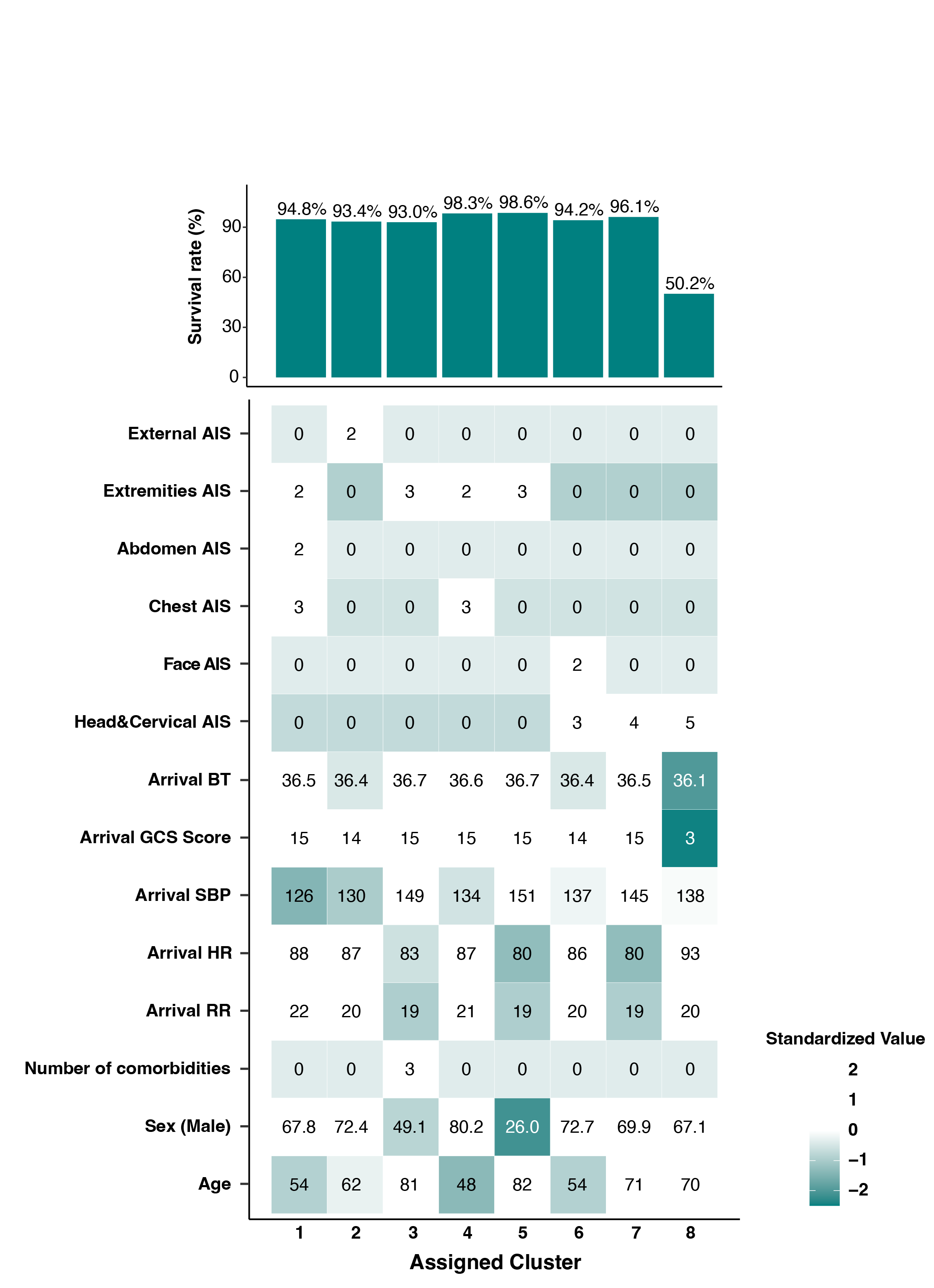
**

**
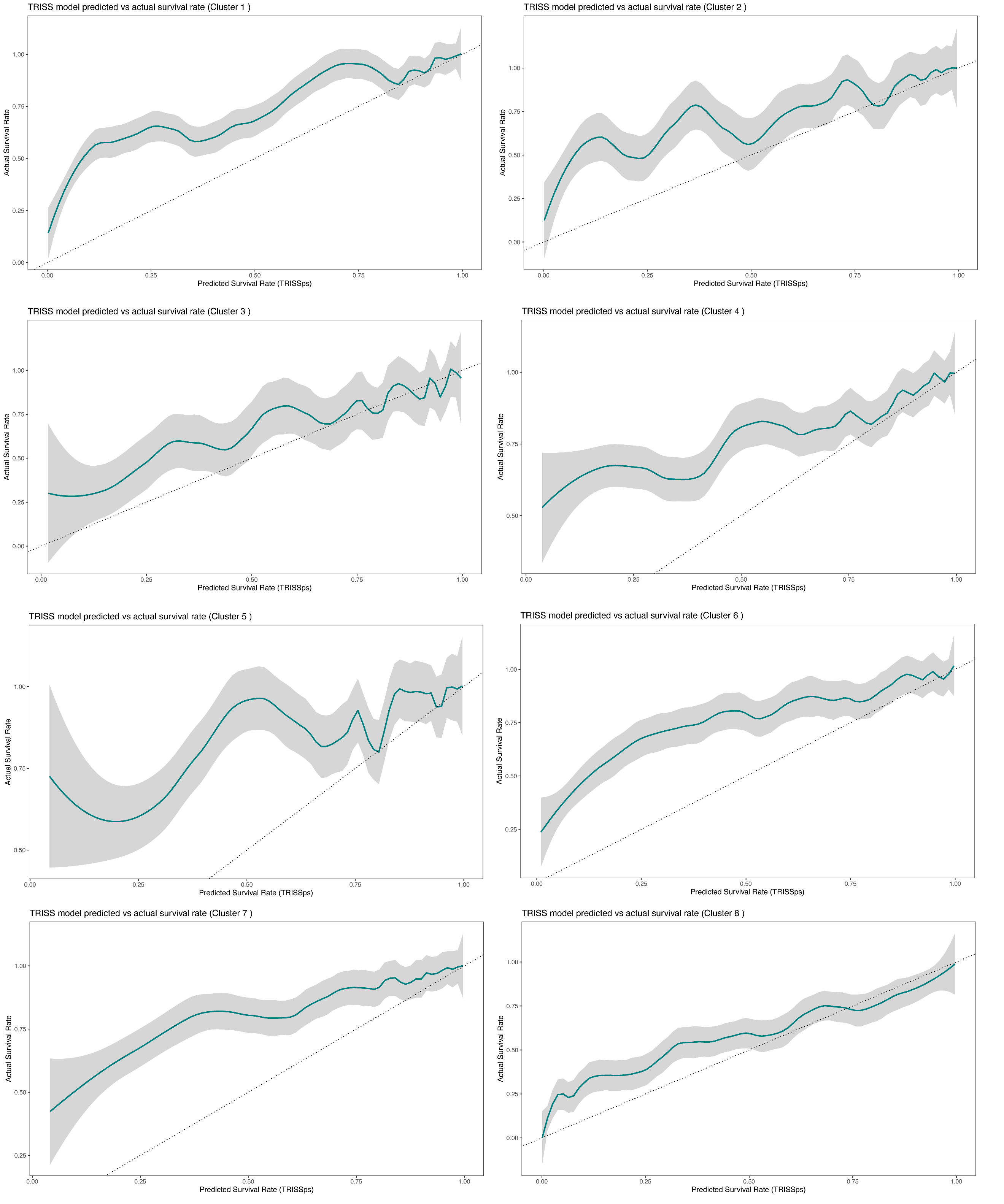
 Figure S7.** Calibration plots showing the relationship between the predicted baseline TRISS model and actual survival rates for each trauma phenotype in the validation cohort (JTDB 2019–2022)

**
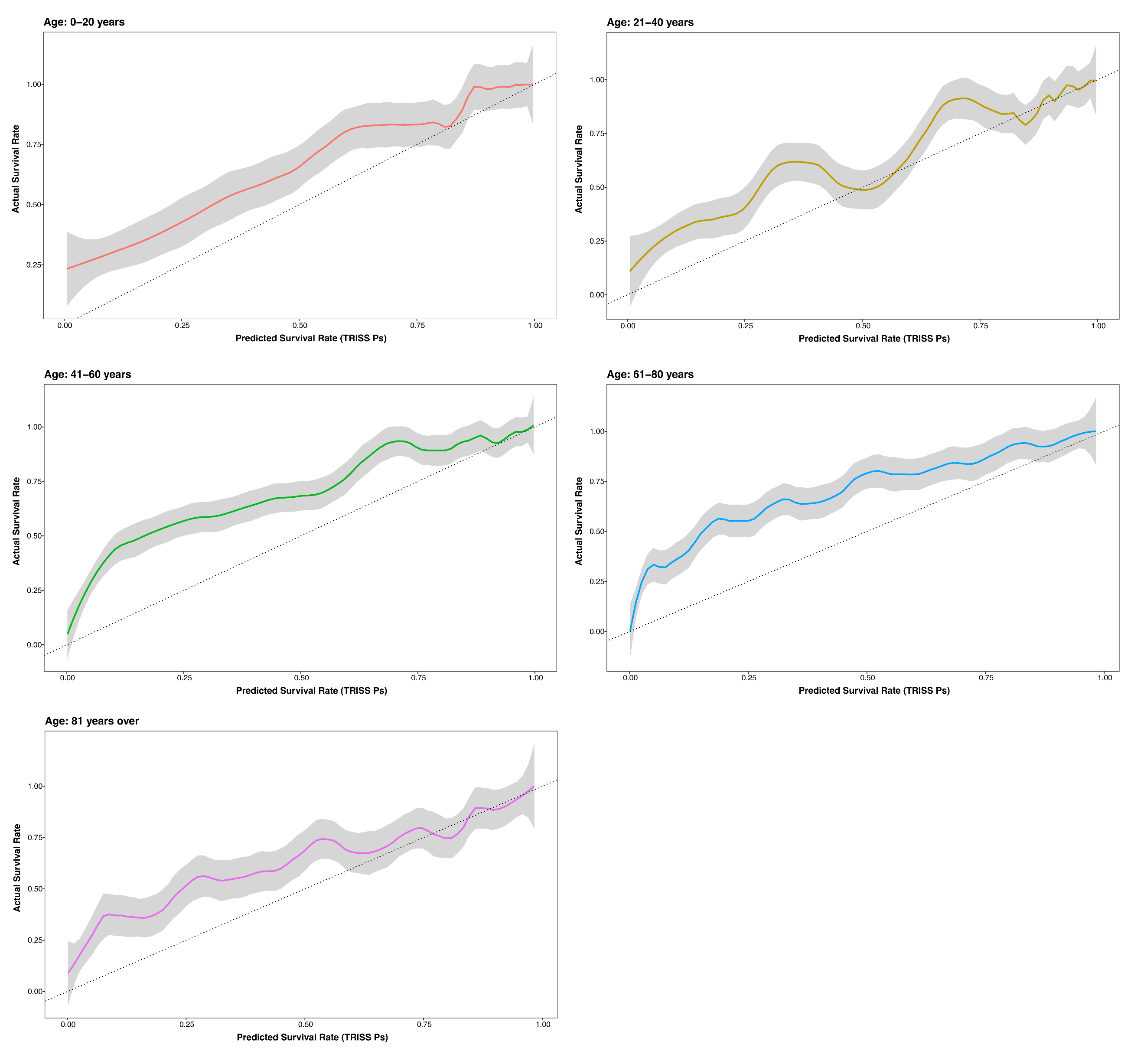
 Figure S8.** Calibration plot of the baseline TRISS model by age categories in the validation cohort (JTDB 2019–2022)

**
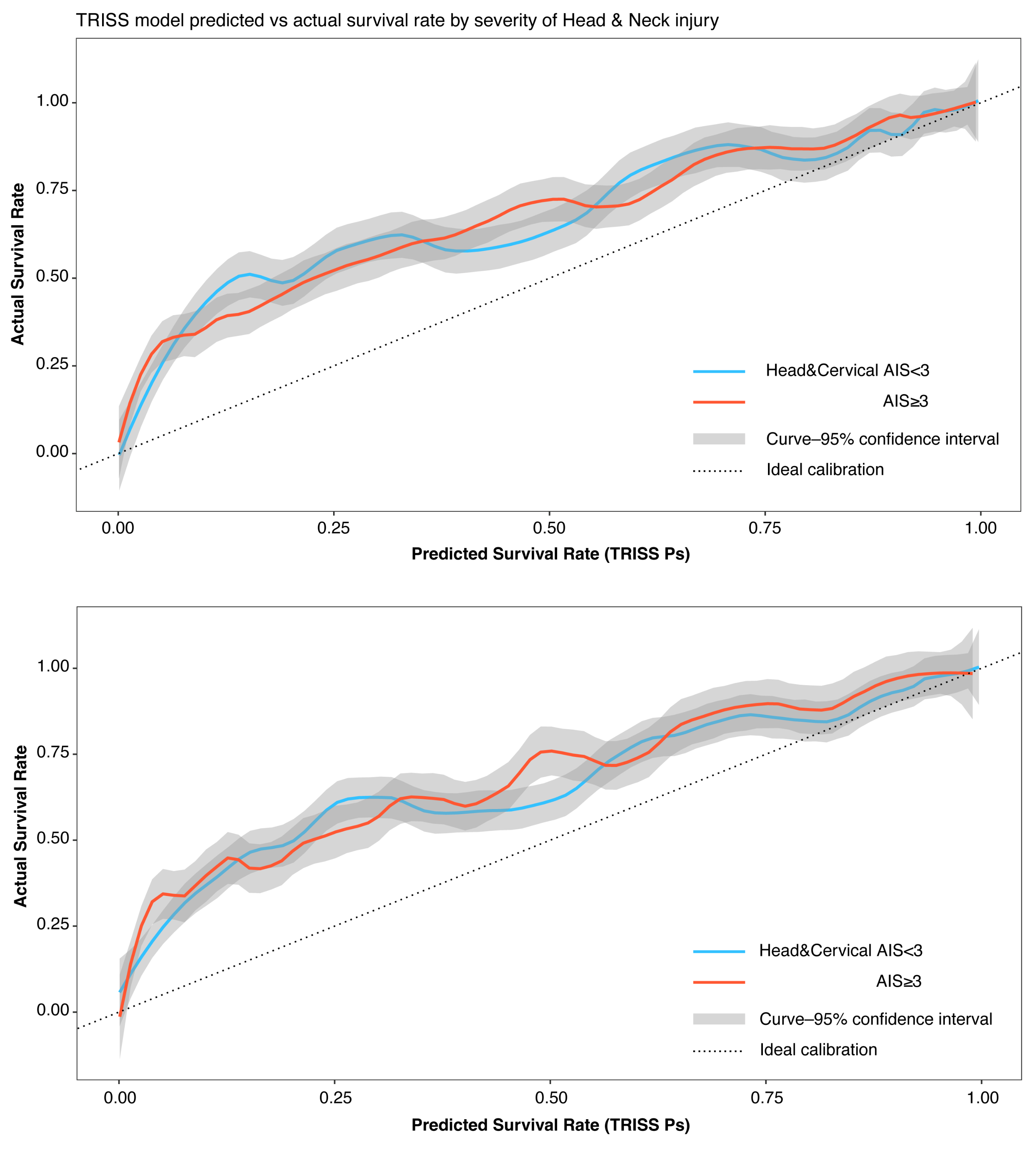
 Figure S9.** Calibration plot of the baseline TRISS model by severity of head and cervical injury (AIS ≥3 vs AIS <3) in the validation cohort (JTDB 2019–2022)

**Figure S10.** Side-by-side calibration plots of the multivariable integrated model specifically for trauma phenotype 8 in the derivation cohort (left) and the validation cohort (right).


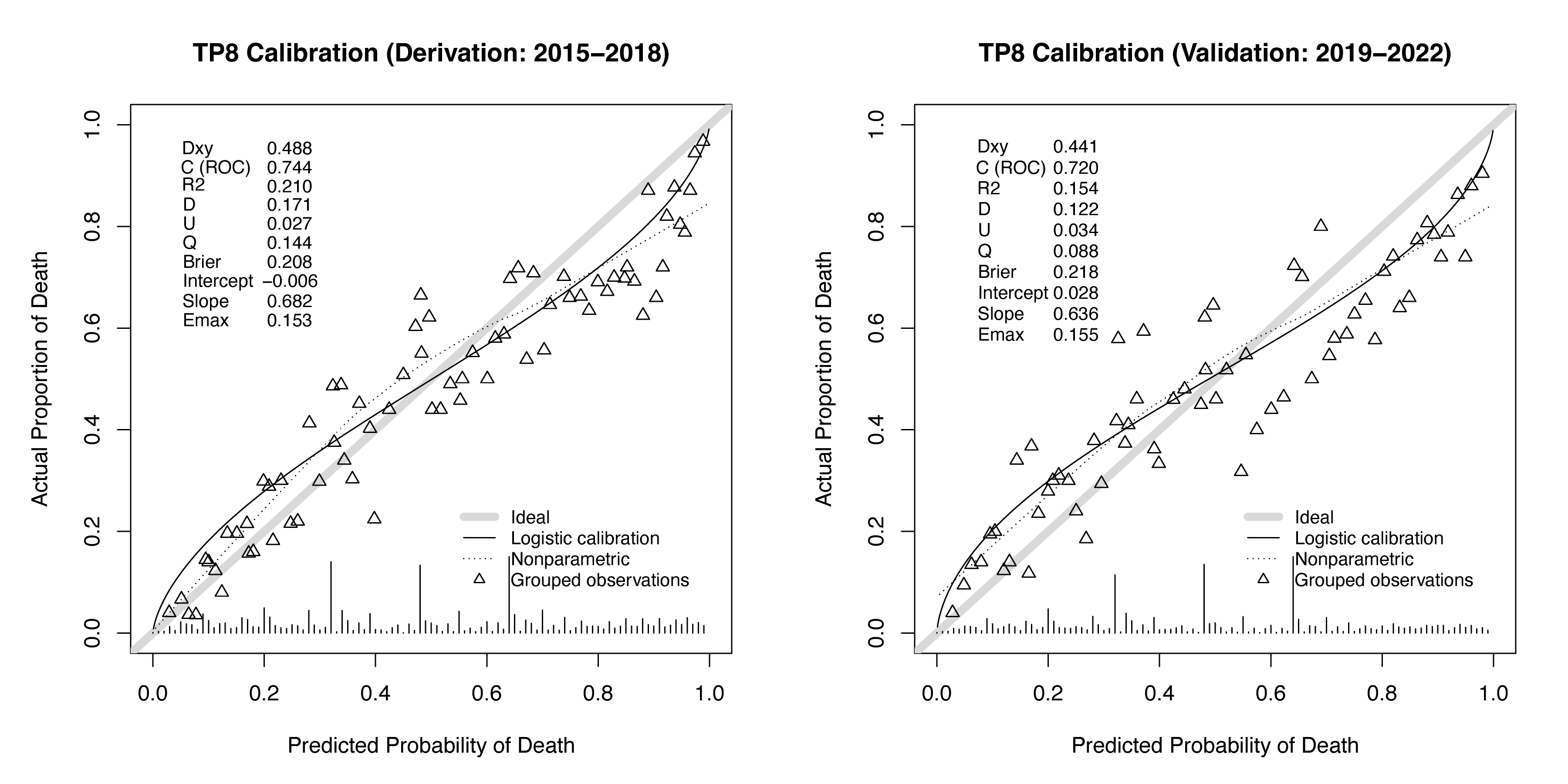


**Figure S1** Workflow diagram detailing the trauma phenotype assignment process and multivariable probability calculation pipeline.

**Figure S2** Patient selection flowchart for the historical derivation cohort.

This flowchart details the inclusion and exclusion criteria applied to establish the derivation cohort for this retrospective cohort study. The cohort consists of patients with blunt trauma registered in the Japan Trauma Data Bank (JTDB) across tertiary emergency medical facilities in Japan between 2015 and 2018. This historical dataset was utilized to evaluate the baseline performance of the TRISS model and to train the integrated multivariable logistic regression model.

ISS, Injury Severity Score; JTDB, Japan Trauma Data Bank

**Figure S3** Correlation matrix heatmap showing the relationships between variables used for phenotype clustering.

**Figure S4** Complex heatmap with the distribution of survival rates and variables for each trauma phenotype in the derivation cohort.

The upper panel shows the survival rates; the lower panel displays the standardised values for each variable. The number in the cells is the median (sex is shown as the percentage of males). AIS, Abbreviated Injury Scale; BT, body temperature; GCS, Glasgow Coma Scale; SBP, systolic blood pressure; HR, heart rate; RR, respiratory rate

AIS, Abbreviated Injury Scale; BT, body temperature; GCS, Glasgow Coma Scale; SBP, systolic blood pressure; HR, heart rate; RR, respiratory rate

**Figure S5** Calibration plots of the TRISS model for each trauma phenotype in the derivation cohort (JTDB 2015–2018).

Each panel represents one of eight trauma phenotypes. The diagonal dotted line represents perfect calibration, whereas the solid line shows the actual relationship between the predicted and observed survival rates. The grey shaded areas indicate 95% confidence intervals.

TRISS, Trauma and Injury Severity Score

**Figure S6** Complex heatmap with the distribution of survival rates and variables for each trauma phenotype in the validation cohort.

The upper panel shows survival rates for each trauma phenotype. The heatmap in the bottom panel shows each variable (standardised and coloured). The number of cells is the median (sex is shown as the percentage of males).

AIS, Abbreviated Injury Scale; BT, body temperature; GCS, Glasgow Coma Scale; SBP, systolic blood pressure; HR, heart rate; RR, respiratory rate

**Figure S7** Calibration plots showing the relationship between the predicted TRISS model and actual survival rates for each trauma phenotype in the validation cohort.

Each panel represents one of eight trauma phenotypes. The diagonal dotted line represents perfect calibration, whereas the solid line shows the actual relationship between the predicted and observed survival rates. The grey shaded areas indicate 95% confidence intervals.

TRISS, Trauma and Injury Severity Score

**Figure S8** Calibration plot of the baseline TRISS model by age categories in the validation cohort (JTDB 2019–2022).

The diagonal line represents perfect calibration. The shaded areas indicate 95% confidence intervals. Age groups: 0–20 years, 21–40 years, 41–60 years, 61–80 years, and ≥81 years.

TRISS, Trauma and Injury Severity Score

**Figure S9** Calibration plot of the baseline TRISS model by severity of head and cervical injury (AIS ≥3 vs AIS <3) in the validation cohort (JTDB 2019–2022). The diagonal line represents perfect calibration. The shaded areas indicate 95% confidence intervals.

AIS, Abbreviated Injury Scale; TRISS, Trauma and Injury Severity Score; TBI, traumatic brain injury

**Figure S10** Side-by-side calibration plots of the multivariable integrated model specifically for Phenotype 8 in the derivation cohort (left) and the validation cohort (right). These plots show the calibration of the multivariable integration for trauma phenotype 8 patients.
