# Supplementary material for: Novel Online Platform for Trauma Care—Integrating Trauma Phenotypes to Optimize the Trauma and Injury Severity Score Model: Retrospective Cohort Study
Source: JMIR Med Inform. 2026 Jun 2;14:e90011. doi: 10.2196/90011 (PMC13273197; doi:10.2196/90011)
Supplement: Multimedia Appendix 2 [file medinform_v14i1e90011_app2.pdf]

Methods

Problem
Accurate trauma outcome prediction remains challenging; the predictive performance of TRISS has declined over time

Validation cohort
(N = 80,964)

Modeling Approach

k-NN clustering &
Multivariable logistic regression

Online platform

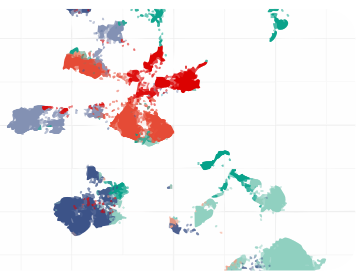

https://github.com/jotarotachino/Trauma-Vis (release v1.0.0)

Early detection

Trauma phenotypes were identified by clustering 14 variables available during initial trauma care

Concept

Integrated multivariable modeling recalibrates baseline TRISS mortality predictions across specific risk profiles.

Model Integration

Covariates: Logit-transformed TRISS
+ 8 Trauma Phenotypes
Model: Multivariable Logistic Regression
Outcome: In-hospital death

Impact

- Significantly improved discrimination (AUROC: 0.889 → 0.897)
- Improved calibration (Brier score: 0.0454 → 0.0394, & LogLoss)
- Higher clinical Net Benefit in decision curve analysis

Integrating trauma phenotypes with TRISS via multivariable modeling significantly improves mortality prediction and clinical net benefit.
